# Disparities in Pain Among Nursing Home Residents: Race, Cognitive Impairment, and Nursing Home Racial Composition

**DOI:** 10.1093/geroni/igaf122.1655

**Published:** 2025-12-31

**Authors:** Cassandra Dictus, Matthias Hoben, Kali Thomas, Tamara A Baker, Baiming Zou, Ruth Anderson, Ashley Leak Bryant, Anna Beeber

**Affiliations:** Duke University, Durham, North Carolina, United States; York University, Toronto, Ontario, Canada; Johns Hopkins University, Baltimore, Maryland, United States; The University of North Carolina at Chapel Hill, Chapel Hill, North Carolina, United States; The University of North Carolina at Chapel Hill, Chapel Hill, North Carolina, United States; Duke University, Durham, North Carolina, United States; The University of North Carolina at Chapel Hill, Chapel Hill, North Carolina, United States; Johns Hopkins University, Baltimore, Maryland, United States

## Abstract

Pain is common among nursing home (NH) residents and can significantly impact quality of end-of-life experiences, yet inequities in pain assessment and treatment persist and are less studied among racial groups with smaller population sizes. This study examined associations between resident race, cognitive impairment, and NH racial composition with any pain documented in the last five days. We conducted a longitudinal multi-level logistic regression using MDS 3.0, LTCFocus, and MBSF data. We focused on residents who died in 2018 or 2019, regardless of place of death (n = 617,922). Analyses were stratified by staff- and self-reported pain, controlling for resident age, function, comorbidity, gender, and NH percent Medicaid, bed number, staffing, ownership, and rurality. In the initial adjusted models, both staff- and self-reported pain increased over the year before death. Staff- and self-reported pain decreased as cognitive impairment increased, and this was more dramatic in the self-reported model. In both models, pain varied by race, with American Indian or Alaskan Native residents having the highest rates, followed by Hispanic and White residents at similar rates; Black residents had slightly lower rates, followed by Asian residents, and Native Hawaiian or other Pacific Islander residents at the lowest. Pain was more frequently reported in predominately White NHs than in more racially diverse NHs, especially in the staff-reported model. Disparities may reflect underassessment of pain and inappropriate pain assessment methods, particularly at the NH-level in facilities that included more racially minoritized residents. Further research is needed to clarify these disparities and ensure equitable pain management.

